# A human mobility dataset collected via LBSLab

**DOI:** 10.1016/j.dib.2023.108898

**Published:** 2023-01-13

**Authors:** Yuwei Zhang, Qingyuan Gong, Yang Chen, Yu Xiao, Xin Wang, Pan Hui, Xiaoming Fu

**Affiliations:** a School of Computer Science, Fudan University, Shanghai 200433, China; b Department of Communications and Networking, Aalto University, Espoo 02150, Finland; c Department of Computer Science, University of Helsinki, Helsinki 00560, Finland; d Department of Computer Science and Engineering, Hong Kong University of Science and Technology, Hong Kong; e Institute of Computer Science, University of Göttingen, Göttingen 37077, Germany

**Keywords:** Mobility data, Location-based services, User check-in, Mood, Weather

## Abstract

Location-Based Services (LBS) have been prosperous owing to technological advancements of smart devices. Analyzing location-based user-generated data is a helpful way to understand human mobility patterns, further fueling applications such as recommender systems and urban computing. This dataset documents user activities of location-based services through LBSLab, a smartphone-based system implemented as a mini-program in the WeChat app. The dataset contains activity data of multiple types including logins, profile viewing, weather checking, and check-ins with location information (latitude and longitude), POI and mood indicated, collected from 467 users over a period of 11 days. We also present some temporal and spatial data analysis and believe the reuse of the data will allow researchers to better understand user behaviors of LBS, human mobility, and also temporal and spatial characteristics of people's moods.

**Specifications Table**

**Table:** 

Subject	Social Sciences
Specific subject area	Mobility, locational trajectories, user-generated data
Type of data	Table
How the data were acquired	The data is collected by a WeChat mini-program from the smartphones of participants as well as a participant questionnaire.
Data format	Filtered
Description of data collection	The user behavioral data was collected from 467 student participants through their smartphone devices when they use the mini-program, *LBSLab*. User demographic information was collected through a questionnaire.
Data source location	• Institution: Fudan University • City/Town/Region: Shanghai • Country: China
Data accessibility	Repository name: A Human Mobility Dataset Collected via LBSLab Data identification number: 10.6084/m9.figshare.15000384.v3 (DOI) Direct URL to data: 10.6084/m9.figshare.15000384.v3

## Value of the Data

• The data contains dynamic check-in data of young smartphone users tagged with spatial and temporal information and rich additional features such as demographic information, mood, POIs, and POI types, as well as the interactions between the users.
• The data can benefit researchers who have interest in human mobility patterns. Analysis of the spatial and temporal patterns of user activities can accelerate the understanding of location-centric behaviors of smartphone users.
• The data can be explored for analyzing and modeling human mobility, profiling users' lifestyles, or studying the connections between user mood and their mobility behaviors.
• The data can be modeled with both predictive and descriptive models. It can be used for tasks including POI recommendation and location prediction.

## 1 Data Description

Location-Based Services (LBS) have become widely used in people's daily life with the convenience provided by smartphones. Particularly, in location-based social networks (LBSNs), users can share their current locations and activities by conducting check-ins at selected points of interest (POIs). They can also share their emotions and life experiences at the locations. These applications record massive data of user activities at different locations. These data provide rich information for researchers to analyze location-related user behaviors [1], [2], [3] (e.g., human mobility patterns and check-in behaviors) with widespread applications (e.g., POI recommendation [4], [5], location prediction [6], [7], traffic prediction [8] and urban computing [9], [10].

This paper introduces a human mobility dataset collected through the *LBSLab* platform. The motivation of creating this dataset is to accelerate our understanding of location-centric behaviors of smartphone users by analyzing the spatial and temporal patterns of user activities.

In this dataset, we include user activity data of 467 student users tagged with locations over a duration of 11 days. The activities recorded include login, checking weather, conducting check-ins, exploring the surroundings, and viewing user ranklist and user profiles. In particular, the check-in data covers user ID, time, accurate user location (indicated by latitude and longitude), tagged POI location along with a corresponding category, and user mood data, providing richer information than traditional check-in records. Besides, we also collected demographic information of the students, including gender and grade, through online questionnaires with their consent. In short, a total of 467 user behavior trajectories including both location-based activities (e.g. conducting check-ins) and social activities (e.g. profile viewing) are included, along with basic user demographic information.

The data is stored in six comma-separated values (CSV) files that are described below in detail.

**Fig. 1:**
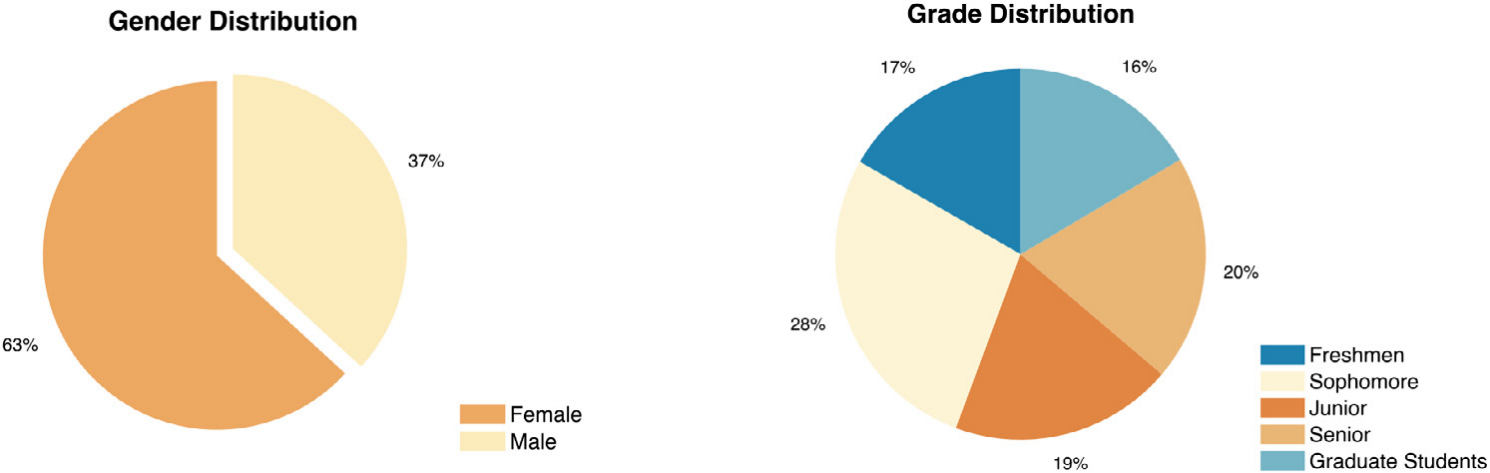
The demographic user data distribution.

The demographic user information collected from the online questionnaires is stored in **user.csv** and formatted as described in Table 1. A copy of the questionnaire is also included in the repository as **questionnaire.pdf**. The data includes the gender and grade of 467 students, with a unique integer generated randomly ranging from 1 to 467 to identify each user. The data distribution is visualized in Fig 1. We can see that the distribution of data is relatively even on the whole, while the number of female participants is slightly larger than male participants. The proportions of students from different grades are basically similar.

**Table 1:** User demographic information is stored in **user.csv** and formatted as described below.

Column name	Column description
user_id	Anonymized ID of the user
gender	Gender of user; 0 for male, 1 for female
grade	Year in college, with 1-4 indicating the grade of an undergraduate student and 5 indicating a graduate student

A description of the user check-in data is available in Table 2 and the data can be found in **checkIn.csv**. There were altogether 20397 check-in records associated with 1931 POIs. Each data record contains the anonymized user ID, day and time information, POI information, and selected mood of a check-in. The *POI_id* here is the same as that obtained from Tencent Maps^1^ and can be used to further look up the detailed information of the POI.

**Table 2:** Check-in records are listed in **checkIn.csv** and formatted as described below.

Column name	Column description
user_id	ID of the user
day	An integer between 1 and 11 indicating the number of the day
day_of_week	An integer between 1-7 representing the day of week, 1 for Sunday and 7 for Saturday
time	Datetime in the form of HH:MM:SS
latitude	Latitude of the location
longitude	Longitude of the location
POI_id	ID of the selected POI on Tencent Map
POI_category	Category of the POI selected, 22 in total
mood	One of five mood types, “happy”, “ordinary”, “sad”, “fearful” and “angry”

**Fig. 2:**
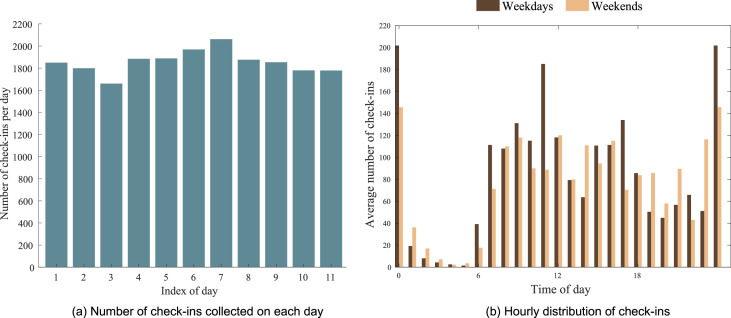
Temporal distribution of check-ins.

Fig. 2(a) summarizes the data availability of check-in actions across the 11 days. As the total number of participants is 467, we can see that each user conducted more than 3 check-ins per day on average.

Fig. 2(b) presents the variation of check-in frequency at different times of the day. The number of check-ins reached the highest at night (12 a.m.) and lowest in the early morning (at 4-5 a.m.). During the day, the number gradually increases from 6 a.m., and there are two peaks at noon and in the afternoon. The data of other user activities also show similar time distribution trends. Such observation is consistent with people's daily patterns. It is common for university students to sleep after 12 a.m., but staying up until 4 a.m. is rare. Also, people tend to take a break from study or work at noon, as well as in the afternoon.

We also compare the patterns between weekdays and weekends. On weekdays, the peaks appeared at 11 a.m. and 5 p.m., when students take lunch and dinner breaks, respectively. In contrast, on weekends there are no such significant peaks by day. Moreover, fewer students got up early at weekends (e.g. the number of check-ins reported at 6 a.m. dropped to half during weekends) and more stayed up late until 2-3 a.m.

**Fig. 3:**
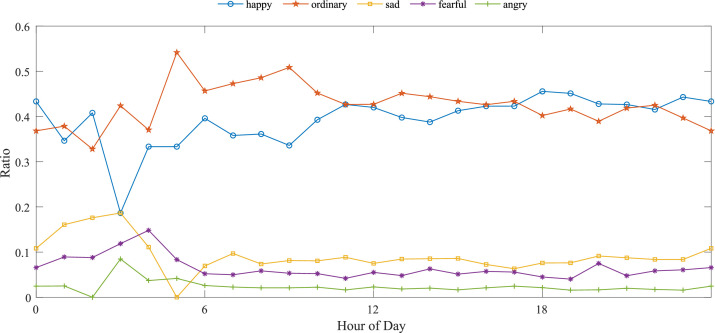
Proportion of user mood trend across the 24 hours.

Next, we verify how temporal characteristics affect users' moods. Fig. 3 shows the percentage of the five moods reported in check-ins over 24 hours of a day. We can see that ``happy'' and ``neutral'' take up most of the users' reported mood, adding up to more than 80 percent in nearly all hours, while negative moods are relatively rare. By and large, the proportion is stable throughout the day. In detail, users' moods are generally neutral in the morning and show an upward trend as the day goes on. The negative emotions are significantly stronger from the mid-night period to the early hours in the morning (1 a.m.-4 a.m.), with ``sad'' and ``fear'' more commonly shown than ``angry''.

**Fig. 4:**
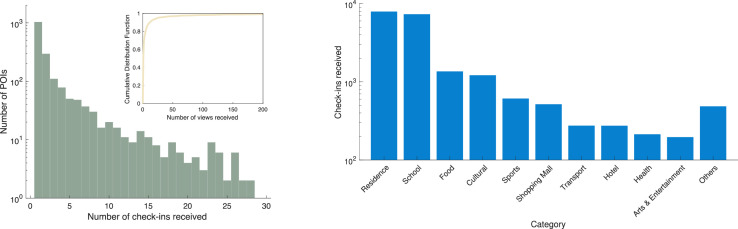
Spatial pattern of the user check-in data.

**Fig. 5:**
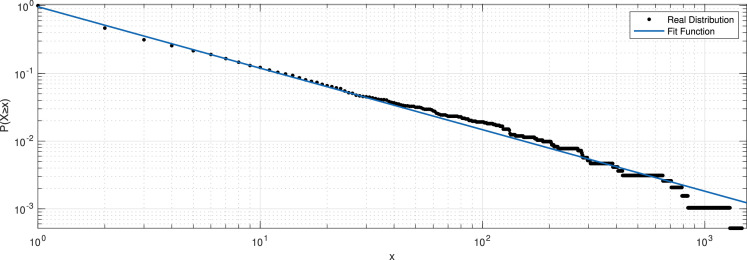
CCDF of number of check-ins per POI.

The spatial properties of the check-in data can serve as a reflection of users' mobility patterns. We counted the number of check-ins at each POI location and show the distribution in Fig. 4 (a). The observed distribution basically conforms to the power-law distribution. $P\left(X\geq x\right)∼x^{-\alpha }$. We optimize the parameters and measured the performance of the fitting using $R^{2}$, coefficient of determination, and get an α of -0.9079 and $R^{2}$ of 0.9951, demonstrating a good fit. It is illustrated in Fig. 5.

The distribution shows that a small portion of the POIs contributed to most of the check-ins, which is consistent with previous studies on check-in data, for example, a study on Foursquare [11] and another on WeChat [12]. This demonstrates that this dataset exhibits typical check-in data characteristics for social networks.

Fig. 4(b) shows the distribution of check-ins in POI categories. Note that the scale of the y-axis is log-based. Most of the check-ins were conducted in the “School” and “Residence” categories, which is consistent with the daily routines of university students. Other popular POI categories include “Food”, “Cultural”, “Sports” and “Shopping mall” venues.

The data records of weather checking, naming POIs and discovering nearby POIs are in **weather.csv, namePOI.csv** and **discover.csv** respectively, which all contain time and location information. The weather field is one of five kinds of weathers, “sunny”, “cloudy”, “overcast”, “foggy” and “light rain”.

The rest of the data records are not related to spatial information. The login data and records of checking the user ranklist are in **login.csv** and **ranklist.csv**. Last but not the least, the data records of profile viewing are in **profile.csv**, which can be interpreted as directional social interactions, with *user_id* referring to the ID of the user conducting the action and *profile_id* indicating the ID of the user whose profile is being viewed

## 2 Experimental Design, Materials and Methods

**Fig. 6:**
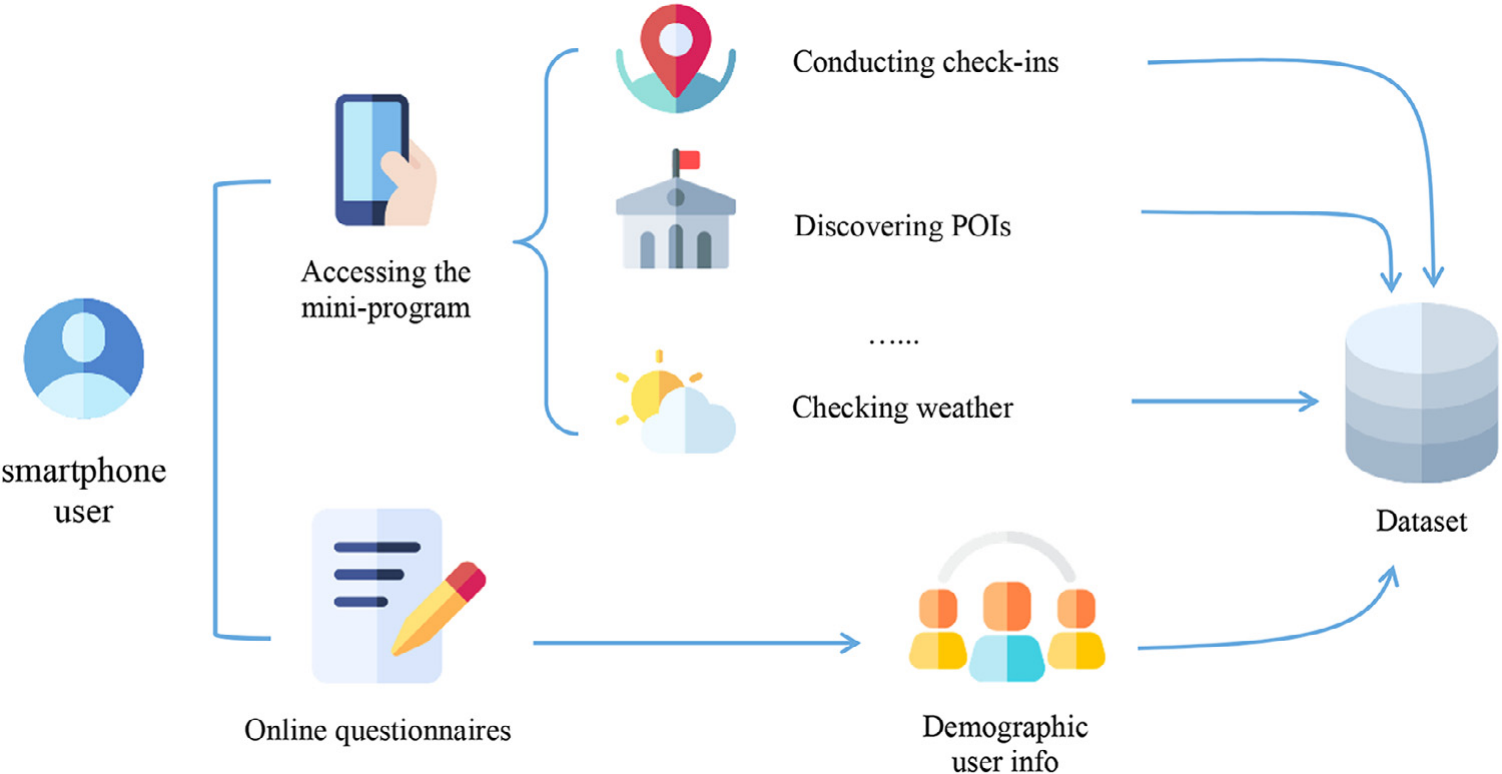
The overall design of the data collection process.

We accumulated the data from two channels, namely location-based behaviors collected via the *LBSLab* platform and demographic information via online questionnaires. Fig. 6 presents the overall design of the data collection system.

In the subsections below, we will introduce the collection methods for both channels in detail.

### 2.1 User activity data collection

We gathered smartphone users' data through LBSLab, a data collection platform built on top of WeChat, a mobile social app that originated in China. Apart from being the most popular messaging and social networking app in China, WeChat also supports mini-programs, a kind of light-weighted sub-applications within the WeChat ecosystem. According to Statista, the number of daily active users (DAU) of WeChat mini-programs in China has reach 440 million by August 2020^2^. In this way, users can easily access LBSLab simply through the WeChat mini-program interface or by scanning a QR code in WeChat. Also, little additional resources like storage space are needed. Meanwhile, making use of WeChat's existing market share and huge user base can also lead to higher user coverage and user activity. Users can easily share the mini-program with their friends via a link, so the rich social network brought by WeChat services is also valuable. Details on the actual implementation of the data collection system can be found in [13], which focused on the design and implementation of LBSLab and also presented a detailed illustration of all the functions provided by the platform.

**Fig. 7:**
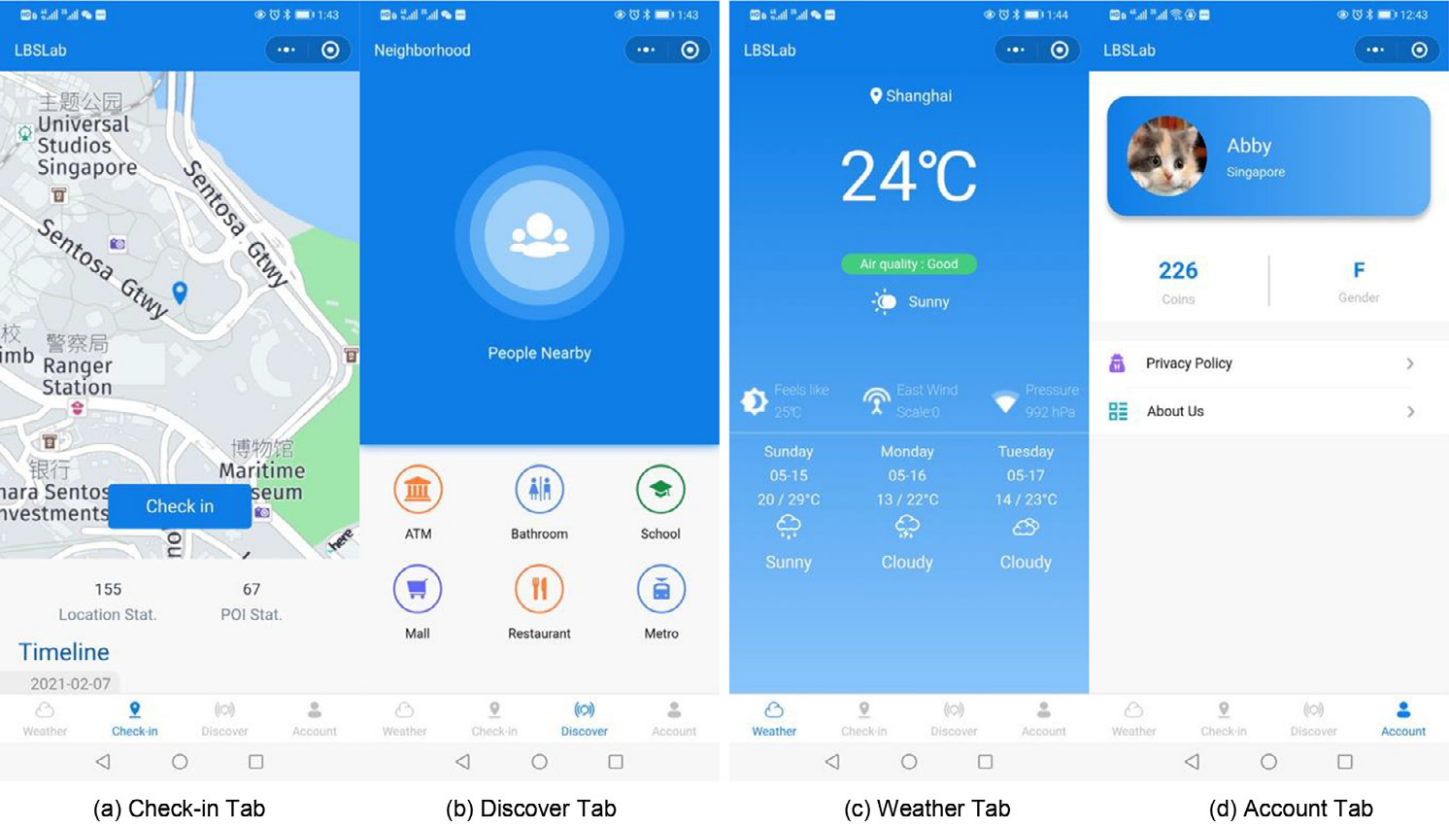
User interface of LBSLab.

LBSLab supports several representative location-based functions, including conducting check-ins at selected POIs, checking the weather of the current location and exploring POIs nearby. Besides, we also implemented a mayorship function to encourage user engagement. Users can grant a nickname to a POI by spending virtual coins received when conducting actions. Users can also check the user ranklist for the top active users as well as view the profiles of other users. The user interfaces of some of the main functions are illustrated in Fig. 7.

We posted advertisements for open recruitment among students in Fudan University and attracted 467 students to participate. After completing the 11-day data collection, each of them was rewarded with 100 CNY.

Locations and timestamps are recorded when users take these location-related actions. The longitude and latitude information is obtained from the official WeChat location API^3^. A series of POIs nearby are then acquired using the longitude and latitude information through Tencent Maps and the user can choose a desirable one to conduct the check-in. As for the weather data, it is obtained using the location data from QWeather^4^ API.

Besides, the actions of logging in and checking the user rank list are also recorded along with time information. Although they do not come with location information, these records can serve as a supplement when analyzing user activities.

We acknowledge that the duration and scale of the data collection is a limitation of this dataset. To fuel user behavior studies on a larger scale, we are planning on collecting data from a larger user pool over longer time periods in the future.

### 2.2 Demographic data collection

We collected demographic information from the 467 university student users with the users' consent, in the form of online questionnaires when they started to use the platform. The information includes the gender and grade of the participants, which are the most basic characteristics of university students.

## Ethics Statements

The aim of this dataset is to enable analysis of location-centric user behavior in the premise of respecting and protecting users' privacy. The user activity records included in this dataset are all public data published by users on the platform, which are open to everyone. In addition to the privacy policy of WeChat itself, each user was explicitly informed of what kind of data will be recorded in detailed privacy terms inside LBSLab, which they agreed with before the data collection.

Also, all users’ identifiers have been anomalized by mapping each to a random ID, and sensitive personal information has been removed. Although it is not impossible to predict user identity based on their mobility traces, it remains a challenging problem because the data is heterogeneous and incomplete [14]. At last, our study was reviewed and approved by the Institute of Science and Technology, Fudan University.

## CRediT authorship contribution statement

**Yuwei Zhang:** Conceptualization, Methodology, Formal analysis, Visualization, Writing – original draft. **Qingyuan Gong:** Conceptualization, Methodology, Project administration. **Yang Chen:** Conceptualization, Project administration, Supervision. **Yu Xiao:** Methodology, Writing – review & editing. **Xin Wang:** Supervision. **Pan Hui:** Methodology, Writing – review & editing. **Xiaoming Fu:** Methodology, Writing – review & editing.

## Declaration of Competing Interest

The authors declare that they have no known competing financial interests or personal relationships that could have appeared to influence the work reported in this paper.

## Data Availability

A Human Mobility Dataset Collected via LBSLab (Original data) (figshare).

## Appendix Supplementary materials

Image, application 1

## Footnotes

1 https://map.qq.com.

2 https://www.statista.com/statistics/1155778/china-number-of-wechat-mini-program-daily-active-users/.

3 https://developers.weixin.qq.com/miniprogram/en/dev/api/location/wx.getLocation.html.

4 https://www.qweather.com.
