# Supplementary material for: A human mobility dataset collected via LBSLab
Source: Data Brief. 2023 Jan 13;46:108898. doi: 10.1016/j.dib.2023.108898 (PMC9898590; doi:10.1016/j.dib.2023.108898)
Supplement: Supplementary file 1 [file mmc1.pdf]

Dear Sir/Madam,

Thank you very much for considering our manuscript DIB-D-22-00668R2. As per request by the editorial office, we hereby provide some information about the ethical issues of this work.

This paper collects some users' data via the WeChat platform. In addition to the privacy policy of WeChat itself, each user was explicitly informed of what kind of data will be recorded in detailed privacy terms inside LBSLab, which they agreed with before the data collection.

Our school does not have an ethical committee, so we are not able to get an "approval number" now (we normally publish papers in IEEE/ACM Transactions, and they never request for such number). Our policy of doing such research is that, we need to file a project proposal first, including the details of the data collection. Then, the Institute of Science and Technology of our university will examine the proposal from various aspects, and such evaluation surely including ethical issues. If the Institute of Science and Technology of our university approves our proposal, which also means that the ethical issues are fine, we could get the university's seal. After that, we could do the proposed research and collect the data we need.

Please refer to the following two pages for our project proposal, including the title page and the seal page. The document is in Chinese, but I have provided translation in the critical information fields.

Thanks again for your great help!

Best regards,
Yang Chen (corresponding author)
School of Computer Science
Fudan University

编号： ISN17- 09

西安电子科技大学
综合业务网理论及关键技术国家重点实验室
开 放 研 究 课 题 合 同 书

State Key Laboratory of Integrated Services Networks
Open Research Project

Massive User Behavior Modeling and
Analysis of Mobile Social Network Users

课题名称： 移动社交网络海量用户行为建模与分析

课题负责人： 陈阳 Yang Chen

课题负责单位： 复旦大学 Fudan University

起止时间：2016 年 1 月至 2017 年 12 月 2016.1-2017.12

通信地址： 上海市张衡路 825 号

邮编 201203 电话 17091929061

负责人电子信箱: chenyang@fudan.edu.cn

2016 年 3 月 21 日

八、合同签署：（请注明该课题有否其它赞助？如有，请注明数额及来源。）该课题无其他资助

乙方：（课题承担方）

Seal of Fudan University

课题负责人（签字）＿＿＿＿＿＿ 2016年3月23日

单位负责人（签字）＿＿＿＿＿＿ 年 月 日

课题承担单位（公章） 年 月 日

开户银行：农行五角场支行营业部

账号：033267-08017003441

甲方（课题委托方）：综合业务网理论与关键技术国家重点实验室（西安电子科技大学）

国家重点实验室负责人（签字）＿＿＿＿＿＿

电话：029-8202524

年 月 日

课题委托单位（公章） 年 月 日

课题依托单位：西安电子科技大学

依托单位负责人（签字）：

年 月 日

*本合同经甲、乙双方签字盖章之日起生效。原项目申请书作为本合同的附件，规定本项目的研究内容和预期成果的细节。
